# Estimating the Development Assistance for Health Provided to Faith-Based Organizations, 1990–2013

**DOI:** 10.1371/journal.pone.0128389

**Published:** 2015-06-04

**Authors:** Annie Haakenstad, Elizabeth Johnson, Casey Graves, Jill Olivier, Jean Duff, Joseph L. Dieleman

**Affiliations:** 1 Institute for Health Metrics and Evaluation, Seattle, Washington, United States of America; 2 University of Cape Town, School of Public Health and Family Medicine, Cape Town, South Africa; 3 Joint Learning Initiative on Faith and Local Communities, Washington, DC, United States of America; London School of Hygiene and Tropical Medicine, UNITED KINGDOM

## Abstract

**Background:**

Faith-based organizations (FBOs) have been active in the health sector for decades. Recently, the role of FBOs in global health has been of increased interest. However, little is known about the magnitude and trends in development assistance for health (DAH) channeled through these organizations.

**Material and Methods:**

Data were collected from the 21 most recent editions of the Report of Voluntary Agencies. These reports provide information on the revenue and expenditure of organizations. Project-level data were also collected and reviewed from the Bill & Melinda Gates Foundation and the Global Fund to Fight AIDS, Tuberculosis and Malaria. More than 1,900 non-governmental organizations received funds from at least one of these three organizations. Background information on these organizations was examined by two independent reviewers to identify the amount of funding channeled through FBOs.

**Results:**

In 2013, total spending by the FBOs identified in the VolAg amounted to US$1.53 billion. In 1990, FB0s spent 34.1% of total DAH provided by private voluntary organizations reported in the VolAg. In 2013, FBOs expended 31.0%. Funds provided by the Global Fund to FBOs have grown since 2002, amounting to $80.9 million in 2011, or 16.7% of the Global Fund’s contributions to NGOs. In 2011, the Gates Foundation’s contributions to FBOs amounted to $7.1 million, or 1.1% of the total provided to NGOs.

**Conclusion:**

Development assistance partners exhibit a range of preferences with respect to the amount of funds provided to FBOs. Overall, estimates show that FBOS have maintained a substantial and consistent share over time, in line with overall spending in global health on NGOs. These estimates provide the foundation for further research on the spending trends and effectiveness of FBOs in global health.

## Introduction

Countless religious institutions and communities are active in public health campaigns and the direct provision of care in low- and middle-income countries (LMICs). This includes faith-based organizations (FBOs), a very diverse category of organizations operating with a mission or vision rooted in religion or faith. Support from governments, community groups, and aid agencies, as well as internal assets, such as committed staff and income generation projects, make the advocacy and work of these institutions possible [1]. Many of these faith-based organizations (FBOs) are well integrated into local communities, having built trust over decades and through the bonds of faith or local ownership [1], [2]. Many FBOs have distinctive characteristics, such as a strong commitment to quality of care and support to rural or otherwise inaccessible communities [2]. Funding entities, such as the World Bank, the Global Fund to Fight AIDS, Tuberculosis, and Malaria (the Global Fund), and the Joint United Nations Programme on HIV/AIDS (UNAIDS), have shown an increased interest in the role of FBOs in developing countries’ health sectors of late, as they have considered the potential contribution and comparative advantage of FBOs in health due to their unique influence and relationship with the communities they serve [1], [3], [4].

The establishment of faith-based health providers (FBHPs; those FBOs directly engaged in the provision of healthcare) can be traced back to the missionaries that accompanied colonizers throughout Asia, Africa, and the Americas. These kinds of FBOs historically had varied funding sources—in early missionary days, funding consisted of a combination of denominational support, donations from international religious communities, and, in some cases, colonial ministry support [3]. Members of religiously motivated communities donated time and money to the causes of hospitals, health clinics, and other actors abroad. Fundraising was tied to religious beliefs, as well as the desire to contribute to health, development, and faith in low-income countries. Broadly speaking, FBOs were funded by organizations and private individuals participating in religious communities [1], [3]. Still today, most FBOs receive the majority of their funding from non-governmental sources. A recent analysis of 76 US international development FBOs shows that only 12.9% of the combined total in revenues was sourced from governments; only 38 received any public funding [5].

Over time there have been changes in the way that some FBOs operate and raise funds. Some FBOs, and especially some FBHPs now receive the majority of their support from a combination of user fees, government grants, and aid agencies. As a share of total contributions, traditional, international denominational bodies are less prominent than in the past [6]. In some LMICs, faith-based nonprofit health facilities have been handed over to local communities and denominations.

There is also massive variation captured within the term “faith-based” [1], [6]. While many FBOs remain small and localized with their local congregations as their main means of support, others are large, professional development organizations that receive substantial development assistance for health (DAH) funding and implement broad-based global health activities. In many cases, FBHPs are now considered part of the national health system, and governments collaborate closely with these faith-based actors. Overall, although some maintain their religiously motivated missions, FBHPs are now commonly classified as part of the development and health sectors.

Due in part to the huge variety of FBOs and the complex funding environment underpinning their operations, gaps in knowledge persist with respect to health-engaged FBOs and their financial support [6]. For example, although FBHPs have played an active role in health in many LMICs for more than a century, there is little solid evidence of exactly how much they contribute to the health systems in these countries. Olivier and Wodon show that most references to FBHPs’ market share can be traced back to data that are over 30 years old [2]. Other estimates fall short in terms of technical rigor, or focus only on select countries, disease areas, or donors [3], [7], [8], [9]. Estimates relying on the number of beds hosted by FBHPs tend to fall short by failing to fully capture utilization across different types of providers and care [3], [10], [11].

Another gap in knowledge relates to the magnitude and nature of the flow of international assistance through FBOs. There is limited historical or current record of how much assistance flows from international religious organizations, development assistance agencies, and even local communities in high-income countries to FBOs in low- and middle-income countries [5]. There are many reasons for this data gap, including historical neglect of research and policy focused on faith-based institutions, and data availability. In addition, many FBOs are reluctant to share financial data with local or international partners [6], [12].

This data gap leaves many questions unanswered. For example, it has been suggested that FBOs might be “disadvantaged” vis-à-vis secular non-governmental organizations (NGOs) in terms of receiving development assistance funding because of bias against their religious nature, or because they continue to work under the radar and outside of global health or development networks [3]. Despite this perception, some limited evidence counters this claim. In South Africa, research shows that for a set of HIV/AIDS-engaged community-based organizations, there was little difference between FBOs’ and secular organizations’ abilities to raise funds [3]. Further research is needed to determine whether these FBOs might be underfunded or not tied into important global health networks.

This research focuses on one component of funding for FBOs: development assistance for health (DAH). DAH is defined as financial and in-kind contributions from global health institutions that aim to improve health in developing countries [13], [14], [15]. We systematically estimate the DAH supplied to a set of FBOs over the last 24 years. For the most part, the FBOs for which we are able to obtain data are channels of DAH. These FBOs play the role of funneling resources sourced from public and private actors in the developed world to implementing agents in developing countries. In some cases, these FBOs can also serve as sources of development assistance, by raising private capital, and in other cases, these FBOs are FBHPs and can also be considered implementing agencies. Still, our focus is on tracking DAH disbursed through FBOs.

Capturing the international funds channeled through FBOs is an important step in understanding the scope of their contributions to global health and how their contributions compare to other NGOs, other development partners acting as channels of DAH, and investments by national governments in their own health systems. Tracking the flow of DAH channeled through the major FBOs provides insight into the types and levels of resources allocated to and disbursed by these organizations, how they leverage public funds in addition to private contributions, and whether the resources available to them differ significantly from other NGOs or other development or public health actors. Knowing the extent to which FBOs are targeted by development assistance partners also exposes trends in global health investments. Better information about FBOs’ involvement in global health could also provide insight into how their comparative advantage could best be harnessed to improve population health in the developing world.

## Material and Methods

Data on the DAH provided by FBOs were collected from three sources used for the *Financing Global Health* DAH database produced by the Institute for Health Metrics and Evaluations (IHME). The annually produced United States Agency for International Development’s (USAID) Report of Voluntary Agencies (VolAg) was the primary resource. This publicly available, annually updated dataset provides information on the domestic and overseas expenditure of private voluntary organizations (PVOs) that received funds from the United States (US) government in a given year, or otherwise voluntarily report to USAID (many NGOs that do not accept public funds still report to the VolAg). To date, this dataset, while focusing to a large extent on US-based NGOs, is the most comprehensive list of NGOs available and includes some NGOs with headquarters outside of the US. The PVOs listed in the VolAg are considered channels of DAH because most are located in developed countries although they conduct their work or channel resources to low- and middle-income countries. Other data were sourced from the Bill & Melinda Gates Foundation (Gates Foundation) and the Global Fund project databases. Data from the Gates Foundation was obtained from the Organisation for Economic Co-operation and Development Creditor Reporting Systems (OECD-CRS) and correspondence, while Global Fund data were downloaded from their online project database. These databases are also updated annually.

As a result, we are able to track DAH through a diverse set of NGOs. Between 1990 and 2010, 1,369 PVOs are listed in the VolAg, with 88.6% based in the US. Table 1 shows that throughout the time series, FBOs made up 26.0–32.6% of all NGOs and, on average, 95.8% of these FBOs were US-based. For these NGOs we have data estimating total expenditure and are able to estimate total DAH channeled through each NGO for each year. In their project-level databases, the Gates Foundation and the Global Fund list 527 and 142 NGOs, respectively. Unlike the NGOs listed in the VolAg, we are only able to track DAH transferred from the Gates Foundation or the Global Fund, not the total DAH expended by each NGO. While several additional aid databases exist, none of them provide comprehensive data on disbursements from NGOs, as NGOs do not report to the OECD-CRS and often report incomplete data to AidData only covering a few of the most recent years.

**Table 1:** NGO composition, 1990–2013.

Year	Total number of NGOs^a^	Number of secular NGOs		Number of faith-based NGOs		Number of US faith-based NGOs		Number of international faith-based NGOs	
1990	268	189	70.5%	79	29.5%	79	100.0%	0	0.0%
1991	340	246	72.4%	94	27.6%	94	100.0%	0	0.0%
1992	391	287	73.4%	104	26.6%	104	100.0%	0	0.0%
1993	419	308	73.5%	111	26.5%	111	100.0%	0	0.0%
1994	439	325	74.0%	114	26.0%	114	100.0%	0	0.0%
1995	430	314	73.0%	116	27.0%	116	100.0%	0	0.0%
1996	434	314	72.4%	120	27.6%	120	100.0%	0	0.0%
1997	441	318	72.1%	123	27.9%	123	100.0%	0	0.0%
1998	496	362	73.0%	134	27.0%	131	97.8%	3	2.2%
1999	493	354	71.8%	139	28.2%	136	97.8%	3	2.2%
2000	510	372	72.9%	138	27.1%	133	96.4%	5	3.6%
2001	530	382	72.1%	148	27.9%	141	95.3%	7	4.7%
2002	569	406	71.4%	163	28.6%	155	95.1%	8	4.9%
2003	595	422	70.9%	173	29.1%	165	95.4%	8	4.6%
2004	601	411	68.4%	190	31.6%	181	95.3%	9	4.7%
2005	596	403	67.6%	193	32.4%	184	95.3%	9	4.7%
2006	629	424	67.4%	205	32.6%	192	93.7%	13	6.3%
2007	650	438	67.4%	212	32.6%	198	93.4%	14	6.6%
2008	699	475	68.0%	224	32.0%	208	92.9%	16	7.1%
2009	722	497	68.8%	225	31.2%	206	91.6%	19	8.4%
2010	714	488	68.3%	226	31.7%	203	89.8%	23	10.2%
2011	714	488	68.3%	226	31.7%	203	89.8%	23	10.2%
2012	714	488	68.3%	226	31.7%	203	89.8%	23	10.2%
2013	714	488	68.3%	226	31.7%	203	89.8%	23	10.2%

^a^The VolAg did not track international NGOs before 1998, resulting in no international faith-based NGOs in the years prior to 1998.

In order to conduct this analysis, a method for determining whether organizations were “faith-based” or not was developed (detailed in S1 Text. Faith-based keywords and screening protocol. and S2 Text. Flow diagram of protocol for hand coding.). There are substantial and unresolved debates on the appropriate classification and identifiers of FBOs [5]. In order to classify organizations as faith-based, we relied primarily on self-identification. Starting with the descriptions in the VolAg, two independent reviewers examined every eligible PVO. In most cases, it was unclear according to the VolAg descriptions whether a PVO was faith-based. In these cases, the reviewers inspected the websites of these organizations, focusing on publicly available mission and vision statements, histories, emblems, and the titles and backgrounds of key personnel. Reviewers designated an organization as faith-based if it:

1. Self-identified as faith-based, mission-based, or as belonging to/being motivated by a particular sect or religious institution;
2. Used keywords (listed in S3 Text. Health-related keywords.) to describe itself or its mission, vision, or projects. For example: religious, Islam, Judaism, Christianity;
3. Referenced working in accordance with holy/religious scriptures, deities, or other religious figures; or
4. Employed members of clergy within a single religious group as 50% or more of staff, leadership, and/or board.

Both reviewers examined 1,947 unique NGOs reporting to the VolAg from 1990 to 2010 or included in the Gates Foundation and Global Fund project databases. (Note, some NGOs were present in multiple databases, thus the total number of NGOs assessed is not the sum of the number of NGOs reported in across three sources of data.) For NGOs listed in the VolAg for more than one year, we reviewed the earliest and most recent NGO descriptions, although we collected financial data for each year. The two independent reviewers had concordant identification for 1798 PVOs, or 92.3% of the sample. For the remaining 149 organizations, final determination was made by a third independent reviewer, and was based on the same classification criteria in addition to discussion with other experts in the field of faith-based health services.

Estimates of the amount of DAH provided via international and US-based FBOs are based on a linear regression model that predicts overseas health expenditure as a fraction of total overseas expenditure. We collected health expenditure data from 990 tax forms, annual reports and audited financial statements for the yearly top 30 NGOs in terms of overseas expenditure and 20 randomly selected NGOs, with the probability of being selected set proportional to overseas expenditure. We then fit a linear regression model to predict health expenditure as a fraction of total expenditure and used this model to predict health fractions for the remaining NGOs. Five of the nine variables in the regression were drawn from the VolAg reports, including the fraction of revenue from in-kind donations, fraction of revenue from the US government, fraction of revenue from private financial contributions, overseas expenditure as a fraction of total expenditure, and calendar year, while the last four variables were binary indicators based on keyword searches of the NGO’s name and description for health-related words and non-health related words. The predicted fractions were then multiplied by total overseas expenditure to estimate total overseas health expenditure. These methods have been peer-reviewed and are applied by IHME in its annual *Financing Global Health* report [14], [15].

In addition to identifying and estimating DAH from PVOs reporting in the VolAg, we delved deeper into the practices of two particular donors: the Gates Foundation and Global Fund. Both organizations provide project-level disbursements that allowed us to estimate the share of their contributions channeled to NGOs through 2011. Both databases provide information on the recipient agency and when the agency was an NGO, we followed the same method as described above to distinguish FBOs.

## Results

Of the organizations listed in the VolAg, 360 (26.3%) were identified as faith-based. In 2013, DAH spending by these organizations amounted to $1.53 billion (in 2011 US dollars). This was a 4.7% increase over 2012, when spending amounted to $1.46 billion. Over the entire 24-year period available, development assistance for health to FBOs that are listed in the VolAg or Gates Foundation and Global Fund databases (FBO DAH) has grown substantially. In 1990, FBO DAH expenditure amounted to $166.3 million. Growth in spending has totaled more than $1 billion since 1990. In annualized terms, FBO DAH has grown 10.1% per year during this period. Year-over-year increases were highest from 1999 to 2008. In recent years, 2011–2013, FBO DAH has not grown as rapidly.

**Fig 1:**
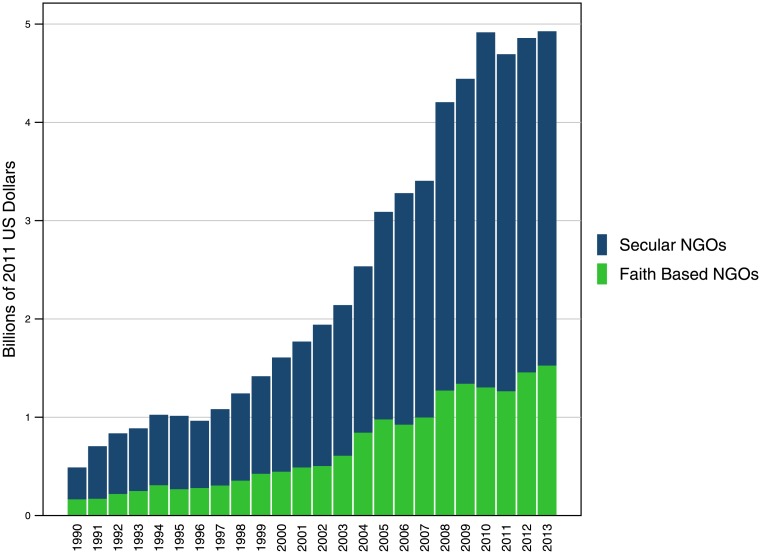
Faith-based spending among all US- and internationally-based NGOs, 1990–2013. DAH spending among all NGOs has increased steadily over time, and expenditure by FBOs has increased at a similar rate. While FBOs’ DAH expenditure has increased over time, the proportion of FBO expenditure, vis-à-vis overall NGO expenditure, has remained consistent.

FBO DAH grew at rates similar to NGO DAH as well as total DAH across all channels. As shown in Fig 1, the share of DAH provided by FBOs, as compared to NGOs overall, has remained relatively steady since 1990. Table 2 shows that in 2013, FBO spending made up 31.0% of total NGO DAH. This is slightly less than 1990 when FBOs spent 34.1% of total DAH provided by NGOs. With respect to the total DAH envelope across all channels, FBO DAH amounted to 4.9% of expenditure. The share has varied over the 1990–2013 period, but not widely or consistently in any direction, ranging from 2.9% to 5.7%. FBO DAH has also kept pace with total DAH growth; from 1990 to 2013, total DAH grew 7.6% annually, while FBO DAH grew at a slightly higher rate of 10.1% annually.

**Table 2:** Total NGO FBO spending, 1990–2013.

Year	Total NGO DAH (billions of 2011 US dollars)	Faith-based NGO DAH (billions of 2011 US dollars)	Secular NGO DAH (billions of 2011 US dollars)	Faith-based share of total
1990	0.49	0.17	0.32	0.341
1991	0.70	0.17	0.53	0.243
1992	0.84	0.22	0.61	0.264
1993	0.89	0.25	0.64	0.283
1994	1.02	0.31	0.71	0.302
1995	1.01	0.27	0.74	0.265
1996	0.96	0.28	0.68	0.292
1997	1.08	0.31	0.77	0.284
1998	1.24	0.36	0.89	0.287
1999	1.42	0.43	0.99	0.301
2000	1.61	0.45	1.16	0.278
2001	1.77	0.49	1.28	0.277
2002	1.94	0.50	1.44	0.260
2003	2.14	0.61	1.53	0.285
2004	2.53	0.84	1.69	0.333
2005	3.09	0.98	2.11	0.317
2006	3.28	0.93	2.35	0.282
2007	3.40	1.00	2.41	0.293
2008	4.20	1.27	2.93	0.303
2009	4.44	1.34	3.10	0.302
2010	4.91	1.30	3.61	0.265
2011	4.69	1.27	3.43	0.270
2012	4.86	1.46	3.40	0.300
2013	4.93	1.53	3.40	0.310

In order to better understand how and where FBOs are expending their DAH, we examined expenditure trends among the top FBOs by DAH. Table 3 shows the ten FBOs channeling the largest amount of DAH in 2013. Food for the Poor tops the list, disbursing $782.4 million in 2013. MAP International, at $352.3 million, took the next slot, followed by Catholic Medical Mission Board, at $252.8 million. At number ten, CitiHope International disbursed $52.9 million in DAH in the same year.

**Table 3:** The top ten largest FBO DAH recipients, 2013.

	DAH (millions of 2011 US Dollars)
Food For The Poor, Inc.	782.41
MAP International, Inc.	352.31
Catholic Medical Mission Board, Inc.	252.79
Catholic Relief Services—USCC, Inc.	244.68
Feed the Children, Inc.	193.38
Northwest Medical Teams International	150.90
Interchurch Medical Assistance, Inc.	148.04
World Vision, Inc.	113.60
Christian Blind Mission International	85.21
CitiHope International, Inc.	52.92

**Fig 2:**
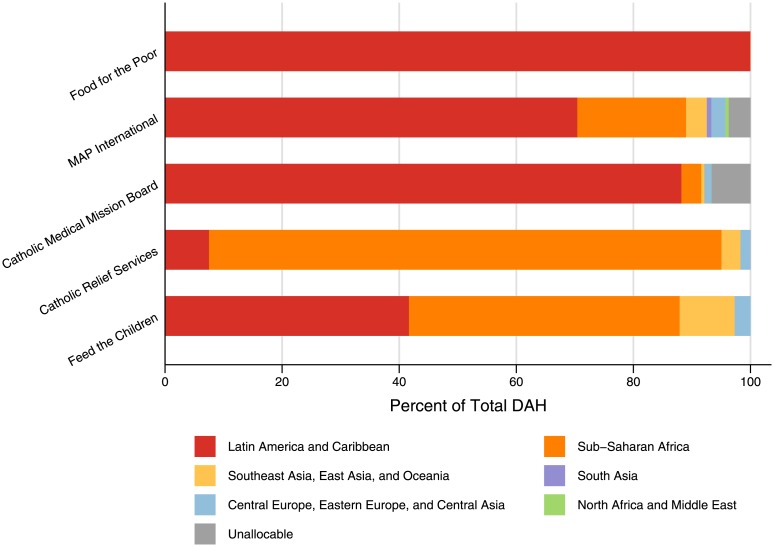
Top five largest FBOs’ DAH expenditure by region, 2013. The top five FBOs by DAH expenditure are Food for the Poor, MAP International, the Catholic Medical Mission Board, Catholic Relief Services, and Feed the Children. The overwhelming majority of their DAH is expended in Latin America and the Caribbean and sub-Saharan Africa. This is in contrast with overall DAH, which has the highest proportion of spending in Asia.

Fig 2 displays the top five FBOs’ DAH spending across regions and shows that these organizations tend to favor sub-Saharan Africa and Latin America and the Caribbean more than other regions. The focus on these regions contrasts with DAH overall, which has a higher proportion of expenditure in Asia. The top five organizations, on the whole, also tend to focus a large portion of their spending on health, with 62.4% of total expenditure going to health on average for the top five. Both MAP International and Catholic Medical Mission, with 99.3% and 98.4%, respectively, spend nearly all their funds on health. However, the shares of spending do vary widely across the top FBOs, including Food for the Poor (67.0%), Feed the Children (39.1%), and Catholic Relief Services (8.0%).

**Fig 3:**
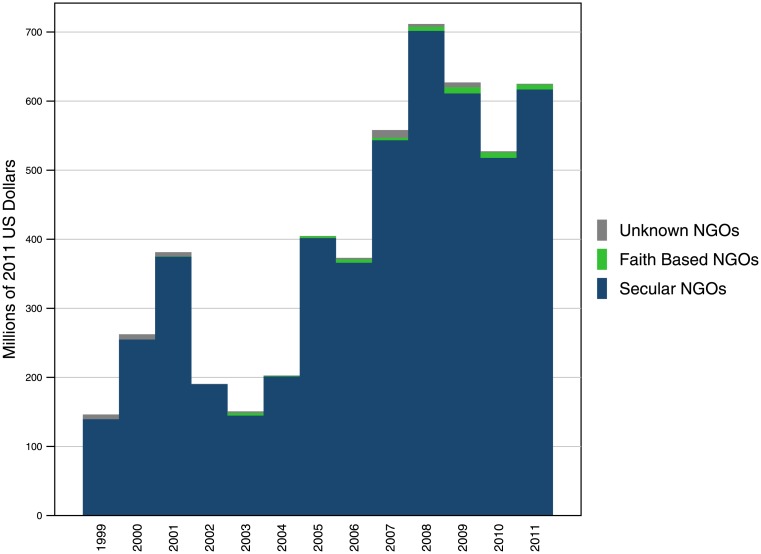
Bill & Melinda Gates Foundation’s expenditure on FBOs, as a portion of NGO spending, 1999–2011. The Gates Foundation has historically funded a small, but steady portion of FBOs. The major recipients are FBHPs, in the form of faith-based hospitals, and a select few NGOs.

The two other channels analyzed, the Gates Foundation and Global Fund, show that contributions to FBOs differ across development assistance partners. As shown in Fig 3, the Gates Foundation has provided a small, but steady share of funds to FBOs. In 2011, the Gates Foundation contributed $7.1 million to FBOs, or 1.1% of the total provided to NGOs overall. Between 2003 and 2011, funds disbursed by the Gates Foundation to FBOs has grown at an annualized rate of 7.7%. As shown in Table 4, much of this expenditure is allotted to FBHPs, such as St. Michael’s Hospital, Emmanuel Hospital Association, PROMETRA International, and the Philippine Lactation Resource and Training Center, as well as Bread for the World. The Global AIDS Interfaith Alliance has also been a major recipient in past years.

**Table 4:** FBO recipients of Bill & Melinda Gates Foundation’s support, 2011.

	DAH (millions of 2011 US dollars)
St. Michael's Hospital	2.73
Emmanuel Hospital Association	2.40
Bread for the World Institute	0.69
PROMETRA International	0.58
Philippine Lactation Resource and Training Center	0.10

**Fig 4:**
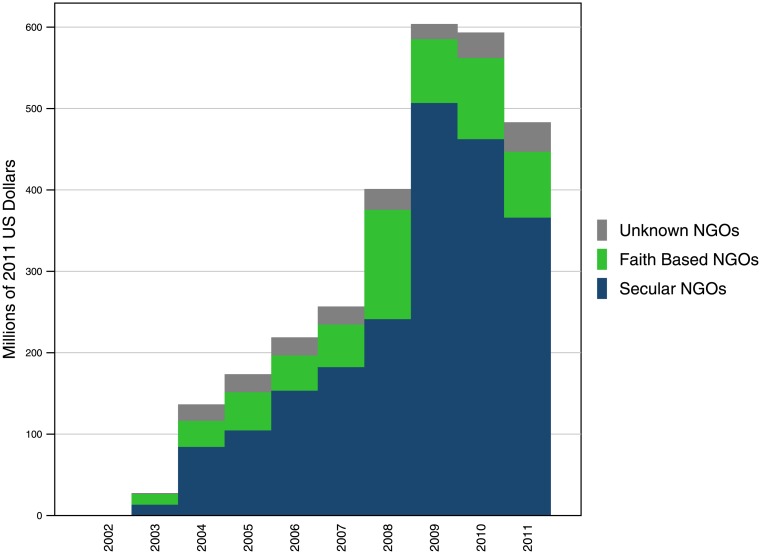
The Global Fund’s expenditure on FBOs, as a portion of NGO spending, 2002–2011. The Global Fund works closely with FBOs, among other local actors, to channel its DAH funds to low- and middle-income countries. Though the share of funds received by FBOs from the Global Fund has drop from its peak in 2008, the Global Fund has provided a substantial greater share of its funds to FBOs in recent years than it did at its inception in 2002.

In 2011, the Global Fund provided $80.9 million to FBOs, as shown in Fig 4. This amounted to 16.7% of total contributions to NGOs. Fig 4 also shows that FBOs received the greatest share of Global Fund expenditure in 2008, at $134.3 million, or 33.5% of total funding to NGOs. Despite the minor drop in funding between 2008 and 2011, the Global Fund has increased its disbursements to FBOs since the organization’s establishment in 2002 overall. Annualized growth from 2003 to 2011 was 25.5%. As shown in Table 5, these funds have reached a wide range of organizations, including Christian Health Associations throughout Sub-Saharan Africa, World Vision country offices, and other actors working on the ground to combat HIV/AIDS, tuberculosis, and malaria.

**Table 5:** FBO recipients of Global Fund support, 2011.

	DAH (millions of 2011 US dollars)
Churches Health Association of Zambia	25.32
World Vision Mozambique	7.69
Ethiopian Interfaith Forum for Development, Dialogue and Action	6.27
Catholic Relief Services—Niger	3.30
Caritas India	3.26
Lanka Jatika Sarvodaya Shramadana Sangamaya	3.09
Adventist Development and Relief Agency	2.81
World Vision Somalia	2.75
Central Board of Aisyiyah	2.68
Catholic Relief Services USCCB—Benin	2.39

In order to explore trends in FBO DAH more in depth, we examined the DAH channeled through FBOs across six health focus areas (HFAs): HIV/AIDS; tuberculosis (TB); malaria; maternal, newborn, and child health (MNCH); non-communicable diseases (NCDs); and “other,” which consists of all health expenditure that does not fit into any of the other five HFAs. Using NGO names and descriptions reported in the VolAg and project descriptions from the Gates Foundation and the Global Fund databases, we were able to allocate fractions of FBO DAH to these HFAs. Disaggregating expenditure illustrates trends in how FBOs are targeting their spending. Because we do not have project-level data for 2012 and 2013, we were not able to allocate DAH to HFAs in these years.

**Fig 5:**
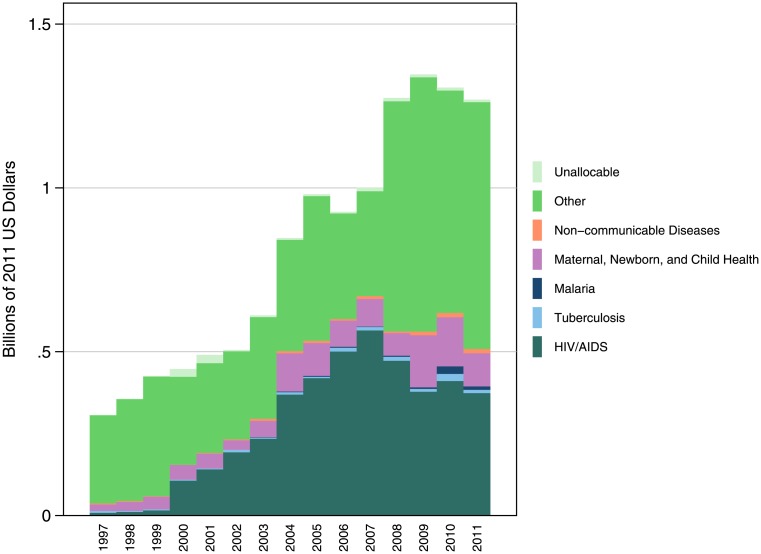
Faith-based spending among all US- and internationally-based NGOs, by health focus area, 1990–2011. Disaggregation of DAH spending among faith-based NGOs has improved of time; the proportion of expenditure going to “other” projects has decreased over the course of the 2000s, as more data for targeting NGOs’ work have become available. The largest share of FBO DAH goes to HIV/AIDS. HIV/AIDS FBO DAH peaked in 2007, and then dropped and leveled off over 2008–2011.

Fig 5 displays the time trends in expenditure by health focus area across FBOs tracked in the VolAg. Between 1999 and 2000, DAH for HIV/AIDS jumped from $15.8 million to $106.5 million, a 572.8% increase. HIV/AIDS DAH by FBOs continued to grow at an annualized rate of 26.9% until its peak in 2007. Over 2008–2011, HIV/AIDS DAH held steady. Only 0.8% of FBO DAH went to malaria in 2011, compared to 5.6% of total NGO DAH. In 2011, shares of FBO DAH and total NGO DAH for MNCH were 7.9% and 11.3%, respectively.

**Fig 6:**
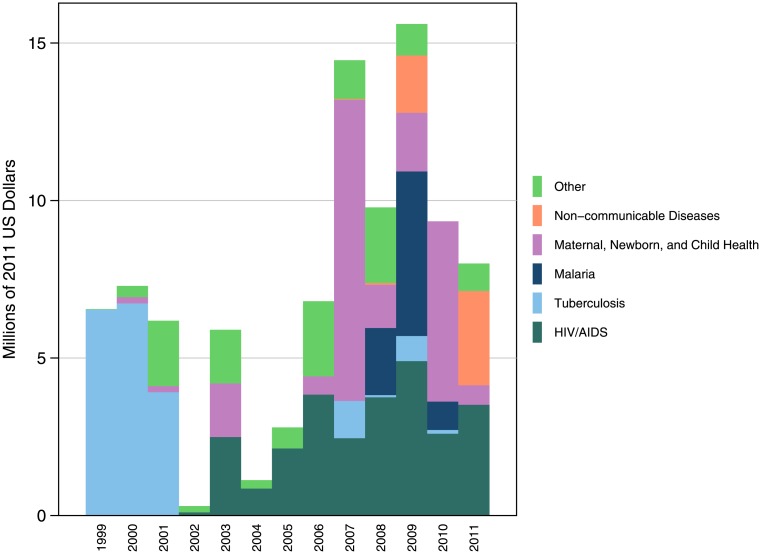
Bill & Melinda Gates Foundation’s expenditure on FBOs, by health focus area, 1990–2011. The Gates Foundation has no consistent funding patterns of how it allocates DAH to FBOs by HFA. In early years, most DAH for FBOs went to TB projects; in recent years, DAH to FBOS targeting HIV/AIDS, MNCH, and NCDs have taken the largest share of DAH.

As Fig 6 shows, the DAH disbursed by the Gates Foundation to FBOs does not follow any real trend with regard to the share of DAH by HFA. For the first 3 years of its existence, the Gates Foundation granted nearly all of its DAH to FBOs supporting TB projects, averaging shares of 87.9%, or $5.7 million, during this period. HIV/AIDS is the HFA with the most consistent funding patterns, but even it varies widely from $102,929 in 2002 to a peak of $4.9 million in 2009. In recent years, funding for NCDs has increased, and was $3.0 million, or 41.1% of total expenditure to FBOs in 2011. This highlights the increasing prevalence of NCDs as a major global health concern.

**Fig 7:**
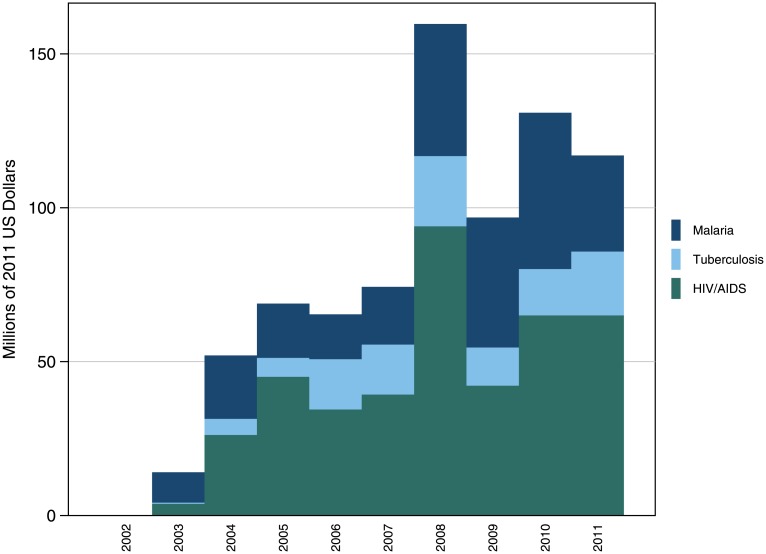
The Global Fund’s expenditure on FBOs, by health focus area, 1990–2011. The Global Fund targets 3 diseases—HIV/AIDS, TB, and malaria. The relative expenditure ratios have remained consistent since 2002, with HIV/AIDS receiving the largest share of expenditure, followed by malaria, and TB receiving the smallest share of expenditure for FBOs.

The Global Fund’s mandate covers just three HFAs: HIV/AIDS, TB, and malaria. As shown in Fig 7, DAH to FBOs for both HIV/AIDS and TB peaked in 2008 at $94.0 million and $22.8 million, respectively. This resulted in a huge spike in DAH to FBOs for that year. All three HFAs enjoyed steady annualized growth between 2003 and 2011, with rates of 42.5% for HIV/AIDS, 64.3% for TB, and 15.6% for malaria. While shares of expenditure have changed over time, the relative relationship between HFAs has remained consistent, with most DAH targeting HIV/AIDS, followed by malaria, and TB receiving the smallest relative share of funds. Expenditure in 2011 was $65.1 million for HIV/AIDS (55.6% of total), $31.2 million for malaria (26.7%), and $20.7 million for TB (17.7%).

## Discussion

Trends in DAH among FBOs that work on health are in some ways similar to trends in DAH and general NGO DAH overall; in other ways they are distinct. On the whole, since 1990, the FBOs tracked via the VolAg, Gates Foundation, or Global Fund databases have maintained a relatively constant share when measured against total DAH as well as the portion of DAH channeled through NGOs. DAH underwent a major expansion from 2000 to 2010 and, concordantly, FBO DAH increases mirrored this trend. Estimates show that FBOs have received a substantial and consistent share of around 30% of total NGO DAH from 1990–2013.

However, the sources of funding and focus of expenditure are different for these organizations. FBOs tend to spend more on field operations (as opposed to research, development, or global policy and advocacy) [3]. They also tend to concentrate on two regions in particular: Latin America and the Caribbean and sub-Saharan Africa.

These characteristics are reflected in the investment choices of key donors, underpinned by the different niches these development assistance partners fill in the global health landscape. Examining the preferences of the Global Fund and the Gates Foundation in particular displays that contrast. The Global Fund typically funds organizations working in the fight against HIV/AIDS, tuberculosis, and malaria on the ground. The Global Fund supports country coordinating mechanisms that, by definition, work with implementing agencies like the FBOs that support the implementing institutions that distribute bed nets or provide anti-retroviral treatment. The Global Fund invests heavily in the delivery of health services, public health campaigns, and other activities that FBOs typically support. In contrast, while still contributing a substantial amount to FBOs, the Gates Foundation does not provide as much in relative terms. While the Gates Foundation also provides funds to on-the-ground activities, it concentrates more of its investments, as compared to the Global Fund, in the research and development side of health. This is an area of global health in which FBOs typically play a less substantial role.

Analysis of the health focus areas funded also highlights key funding decisions among FBOs as compared to other NGOs. A majority of FBO expenditure (60.0%) falls into the “other” expenditure category. This contrasts with the other share of general NGO spending, which typically amounts to less than 45%. Overall, FBOs are less likely to be focused on the core health focus areas.

Additionally, the large variation in expenditure levels, as provided by the Gates Foundation and the Global Fund, illustrates inconsistencies in funding flows to FBOs. The health focus area supported by the Gates Foundation, in particular, vary substantially from year-to-year. Expenditure on maternal, newborn and child health was null in 2004 and 2005, for instance, but made up 70.8% of all Gates Foundation spending on FBOs in 2007. In contrast, the Global Fund’s spending generally maintained a consistent distribution across areas, but the spike in 2008 represents a shock of spending that was not maintained. Overall, these one-time injections of funds for FBOs in certain health focus areas raises questions about the sustainability and predictability of the DAH provided to FBOs.

The data sources used provide the most comprehensive list of NGOs currently available. However, clear limitations are associated with the scope of coverage. The data focus exclusively on public funding for FBOs. With taking private funds into account, total expenditures of FBOs may be significantly underestimated. Furthermore the VolAg dataset captures primarily US-based FBOs. This means our list captures channels, not recipients, of DAH. This could bias the list against small organizations operating domestically in developing countries or organizations that predominately receive funds from other developed country governments. Tracking mainly US-based NGOs may also introduce religious bias, with the majority of FBOs being Christian-based. Our analysis may not capture the work of other denominations of FBOs, operating in different parts of the world. Regarding the Global Fund project-level database specifically, we track principal grant recipients rather than sub-grantee organizations that work collaboratively with the principal recipients. Thus, if the Global Fund channels DAH through a non-faith-based NGO but the NGO channels these resources through a FBO, it is possible that we are not fully accounting for Global Fund DAH channeled through FBOs. Another limitation is that our analysis relies on organizations publicly pronouncing their faith in the VolAg or on their website. It is possible that some organizations may not be forthcoming about their religious affiliation. However, we maintain that a core reason FBOs are of interest is because of donors’ and communities’ knowledge of their affiliation.

The data limitations of this analysis highlight the challenges that arise from poor data and the need for more research on the role of FBOs in funding global health. This analysis highlights the increasing importance of FBOs in global health financing and the shift in the amount of resources available to them. However, it is impossible from this research to conclude whether or not FBOs perform better than other channels in delivering DAH. This research explores the historic trends and current status of FBO DAH, thereby laying the foundation for future analysis of the effectiveness of FBOs in disbursing health aid vis-à-vis other donor channels and national governments.

## Conclusion

Overall, this analysis shows that the evolution of FBO DAH corresponds well with the major expansion in total DAH observed over the last two decades. However, the distinct characteristics that allow FBOs to fill a particular niche in health may make them vulnerable to changes in the spending patterns of certain development assistance partners. If the preferences of funders shift or the financing of these donors dries up, this could have consequences for this set of international health actors. FBOs, and the groups that support their work internationally, should stay attuned to shifts in preferences among major donors, particularly those that fund their work, to prevent spending gaps and ensure sustainability in the delivery of health services.

Estimates of DAH provided by a set of faith-based organizations are a step toward understanding how the sector operates on a global scale. These estimates provide the foundation for further research into the spending trends and effectiveness of FBOs in global health. More information is needed about which health sectors FBOs are most active in, and how smaller and non-US based NGOs receive and channel DAH. Furthermore, more research and analysis are required to fully evaluate the role of different varieties of FBOs in global health.

## Supporting Information

S1 Text Faith-based keywords and screening protocol. (DOCX)

S2 Text Flow diagram of protocol for hand coding. (DOCX)

S3 Text Health-related keywords. (DOCX)
