# Supplementary material for: Estimating the Development Assistance for Health Provided to Faith-Based Organizations, 1990–2013
Source: PLoS One. 2015 Jun 4;10(6):e0128389. doi: 10.1371/journal.pone.0128389 (PMC4456102; doi:10.1371/journal.pone.0128389)
Supplement: S1 Text — (DOCX) [file pone.0128389.s001.docx]

### S1 Text. Faith-based keywords and screening protocol.

The screening process proceeded in a series of steps to classify organizations. First, the 120-word descriptions in the VolAg were reviewed according to criteria 1-3 (listed in the Methods section). If any of these components were present, the organizations was designated as a FBO. However, most organizations did not have sufficiently detailed information in their VolAg descriptions to make this determination. Some organizations also did not have descriptions. For these organizations, reviewers proceeded to step 2, which involved reviewing each organization’s website, when available. Websites were located through listings in the VolAg or the Guidestar online database ([www.guidestar.org](http://www.guidestar.org)), or by doing a Google search of the organization’s name as it appeared on the VolAg list. When this was unsuccessful, abbreviations or known alternate names were used. Websites were confirmed by matching the address or location as listed in the VolAg or other identifying information available in the VolAg. Once the website was ascertained, reviewers examined the homepage and any available “about” pages, “history”/“story” pages, “team” pages, “our work” pages, or similar pages for evidence of criteria 1-4 (listed in the Methods section). The reviewers categorized as FBOs those organizations that met any of these criteria. Other organizations were designated as not faith-based. If the organization’s website could not be located or there was little confidence that the website found represented the NGO under consideration, the organization was marked as missing and was not considered in the analysis.

| Adventist | Congregation | Islam/Islamic | Mission | Religious |
| --- | --- | --- | --- | --- |
| Allah | Coptic | Jain | Missionaries | Salvation |
| Amish | Daoism | Jainism | Missionary | Samaritan |
| Anglican | Dutch Reformed | Jehovah’s Witness | Mohammed | Seventh-Day Adventist |
| Baha’i | Ecumenical | Jesuit | Mormon | Shia |
| Baptist | Episcopal | Jesus | Mosque | Shiite |
| Bible | Evangelical | Jewish | Muhammad | Shi’ite |
| Buddhism | Evangelism | Jews | Mullah | Shinto |
| Buddhist | Evangelist | Judaism | Muslim | Sikh |
| Caliph | Faith | Koran | Orthodox | Sikhism |
| Catholic | Faith-based | Latter-Day Saint | Pentecostal | Sunni |
| Christ | God | Lord | Presbyterian | Synagogue |
| Christian | Hindu | Lutheran | Protestant | Torah |
| Christianity | Hinduism | Mennonite | Quaker | Wahhabi |
| Church | Holy | Methodist | Quran | Zion |
| Church of Christ (Scientist) | Imam | Ministries | Qur’an | Zionism |
| Confucianism | Interfaith | Ministry | Religion | Zionist |
