# Supplementary material for: Estimating the Development Assistance for Health Provided to Faith-Based Organizations, 1990–2013
Source: PLoS One. 2015 Jun 4;10(6):e0128389. doi: 10.1371/journal.pone.0128389 (PMC4456102; doi:10.1371/journal.pone.0128389)
Supplement: S2 Text — (DOCX) [file pone.0128389.s002.docx]

### S2 Text. Flow diagram of protocol for hand coding.

Compiled list of NGOs

n = 1,947

Faith-based NGOs

n = 177

Non-faith-based NGOs

n = 1,770

C. Faith-based NGOs

n = 222

Non-faith-based NGOs

n = 1,538

B. Faith-based NGOs

n = 10

Non-faith-based NGOs

n = 1,760

A. Faith-based NGOs

n = 161

Non-faith-based NGOs

n = 16

**Earliest VolAg description:**

**Most recent VolAg**

**description:**

**Website:**

**Any NGO resulting in box A, B, or C is classified as faith-based.**
