# Supplementary material for: Estimating the Development Assistance for Health Provided to Faith-Based Organizations, 1990–2013
Source: PLoS One. 2015 Jun 4;10(6):e0128389. doi: 10.1371/journal.pone.0128389 (PMC4456102; doi:10.1371/journal.pone.0128389)
Supplement: S3 Text — (DOCX) [file pone.0128389.s003.docx]

**S3 Text. Health-related keywords.**

**Health-related:** health, hiv, aids, nutrition, medical, cancer, gavi, gfatm, vaccine, malaria, bednet, ncd, doctor, medicine, medisend, pathologist, lung, physician, tuberculosis, injuries, noncommunicable, paho, syndrome, retroviral, tb, dots, polio, tobacco, smoking, leprosy, eye, blind, pediatric, fistula, population, santé, medecin, pharmaciens, pharmacy, handicap, prosthetics, mariestopes

**Non-health-related:** w

ater, sanitation, agriculture, climate, environmental, torture, forest, orphan, fauna, flora, nature, tree, wildlife, emergency, energy, soybean, book, earth, green, transportation, road, economic, zoological, humanitarian, humane society, food
